# Identification and characterization of a novel distinct *Pi*neal *N*eoplasm with *N*euroendocrine and *E*pithelial *D*ifferentiation (PINNED)

**DOI:** 10.1007/s00401-026-03111-5

**Published:** 2026-10-26

**Authors:** Helena Bode, Abigail K. Suwala, Brian Gudenas, Alicia Fürst, Neal Geisemeyer, Lea Altendorf, Karoline Hack, Caterina Giannini, Ziedulla Abdullaev, Sebastian Brandner, Jan Beckervordersandforth, Matija Snuderl, Josefine Radke, Carlos Rodríguez-Antolín, Chiara Villa, Catherine Godfraind, Lindsey Lowder, Aaron Mammoser, Tyler J. Kenning, Kay Hertel, Michael Brodhun, Rüdiger Gerlach, Christian Mawrin, Guntis Karelis, Paul Kerbs, Isabell Bludau, Andreas von Deimling, Stefan M. Pfister, Felix Sahm, Alexandre Vasiljevic, Christian Thomas, Martin Hasselblatt, Paul Northcott, Marcel Kool, Ulrich Schüller

**Affiliations:** 1 Research Institute Children’s Cancer Center Hamburg, Martinistraße 52, N63, 20251 Hamburg, Germany; 2 Department of Pediatric Hematology and Oncology, Heidelberg University, Heidelberg, Germany; 3 Pediatric Hematology and Oncology, University Medical Center Hamburg-Eppendorf, Hamburg, Germany; 4 Hopp Children’s Cancer Center (KiTZ), Heidelberg, Germany; 5 Division of Pediatric Neurooncology, German Cancer Consortium (DKTK) and German Cancer Research Center (DKFZ), Heidelberg, Germany; 6 Department of Neuropathology, Institute of Pathology, Heidelberg University, Heidelberg, Germany; 7 Clinical Cooperation Unit Neuropathology, German Cancer Research Center (DKFZ), German Consortium for Translational Cancer Research (DKTK), Heidelberg, Germany; 8 Department of Developmental Neurobiology, St. Jude Children’s Research Hospital, Memphis, TN USA; 9 Department of Laboratory Medicine and Pathology, Mayo Clinic, Rochester, MN USA; 10 Laboratory of Pathology Clinical Methylation Unit, Center for Cancer Research, National Cancer Institute, Bethesda, MD USA; 11 Department of Neurodegenerative Disease, UCL Queen Square Institute of Neurology and Division of Neuropathology, University College London Hospitals NHS Foundation Trust, London, UK; 12 GROW-School for Oncology and Reproduction, Maastricht University, Maastricht, the Netherlands; 13 Department of Pathology, Maastricht University Medical Center, Maastricht, the Netherlands; 14 Department of Pathology, New York University Langone Health, New York, NY USA; 15 Institute of Pathology, University Medicine Greifswald, Greifswald, Germany; 16 Institute of Molecular Genomics, University Medicine Greifswald, Greifswald, Germany; 17 Cancer Epigenetics Laboratory, INGEMM, La Paz University Hospital, Biomarkers and Experimental Therapeutics in Cancer, IdiPAZ, Madrid, Spain; 18 Bioinformatics Unit, INGEMM, La Paz University Hospital, Madrid, Spain; 19 CNRS UMR8104, INSERM U1016, Institut Cochin, Université Paris Cité, 75014 Paris, France; 20 Department of Pathology, Hôpital Lariboisière, Assistance Publique-Hôpitaux de Paris, 75010 Paris, France; 21 Neuropathology Unit, University Hospital of Clermont-Ferrand and UMR1071, Clermont Auvergne University, Clermont-Ferrand, France; 22 Department of Pathology and Laboratory Medicine, Emory University School of Medicine, Atlanta, GA USA; 23 Department of Neuroscience and Oncology, Piedmont Brain Tumor Center, Piedmont Atlanta Hospital, Atlanta, GA USA; 24 Neurosurgery Department, Piedmont Atlanta Hospital, Atlanta, GA USA; 25 Department of Pathology and Neuropathology, Helios Clinic Erfurt, Health and Medical University, Erfurt, Germany; 26 Department of Neurosurgery, Helios Clinic Erfurt, Health and Medical University, Erfurt, Germany; 27 Department of Neurosurgery, Helios Klinikum, Erfurt, Germany; 28 Institute for Neuropathology, Magdeburg, Germany; 29 Department of Neurology and Neurosurgery, Rīga Stradiņš University, Riga, 1038 Latvia; 30 Research Professor (Tenured Professor) Group in Neuroimmunology in the Department of Biology and Microbiology of the Faculty of Medicine, Rīga Stradiņš University, Riga, 1007 Latvia; 31 National Center for Tumor Diseases (NCT), Heidelberg, Germany; 32 Department of Pathology and Neuropathology, Groupement Hospitalier Est, Hospices Civils de Lyon, Bron Cedex, France; 33 Institute of Neuropathology, University Hospital Münster, Münster, Germany; 34 Princess Máxima Center for Pediatric Oncology, Utrecht, Netherlands; 35 Institute of Neuropathology, University Medical Center Hamburg-Eppendorf, Hamburg, Germany

**Keywords:** Pineal gland, Pineal tumor, Neuroendocrine, Epithelial differentiation

## Abstract

Pineal region tumors are rare neoplasms that occur in all ages and account for less than 1% of all central nervous system (CNS) tumors in adults but are more frequent in children. Here, we describe a previously unrecognized tumor type of pineal origin characterized by a unique and consistent DNA methylation profile that does not match any known CNS tumor class and is distinct from all currently recognized pineal tumor entities. The presented cohort (*n* = 27) displayed a balanced sex distribution with a mean patient age of 35 years (range 7–75 years). The histopathological analyses demonstrated a highly cellular tumor showing various degrees of necrosis, mitotic activity, papillary structures, rosettes, epithelioid cytology, or signet-ring cells. The tumors consistently expressed cytokeratin 18, synaptophysin, chromogranin A, and OTX2, whereas CRX expression was absent. Four out of 16 tumors (25%) showed *ATRX* mutations and/or loss of protein expression as revealed by immunohistochemistry. Single-nucleus RNA sequencing confirmed molecular distinctiveness from other pineal neoplasms but revealed the closest similarities to papillary tumors of the pineal region (PTPR). Projection of the transcriptome data onto the cellular trajectory of the developing murine pineal gland suggests that pinealocyte progenitors are the cellular origins. Taken together, our findings define a novel type of pineal region tumor that we provisionally named ‘*Pi*neal *N*eoplasm with *N*euroendocrine and *E*pithelial *D*ifferentiation’ (PINNED). Recognition of this tumor type appears to be important for the accurate diagnosis of pineal region tumors and may have implications for future tumor classification and treatment strategies.

**Supplementary Information:**

The online version contains supplementary material available at https://doi.org/10.1007/s00401-026-03111-5.

## Introduction

The pineal gland is a neuroendocrine organ located in the epithalamus and primarily responsible for the production and secretion of melatonin, which regulates the circadian rhythm [1, 4]. During development, orthodenticle homeobox genes, including *OTX2*, play an essential role in pineal development and regulate the expression of *CRX*, a transcription factor involved in pinealocyte differentiation [12, 17, 30, 32].

Pineal region tumors represent a rare group of neoplasms, accounting for approximately 3–11% of pediatric brain neoplasms, while constituting less than 1% of intracranial central nervous system (CNS) tumors in adults [10, 25]. The 2021 World Health Organization (WHO) classification recognizes five distinct tumor entities of the pineal region: pineocytoma (PC), pineal parenchymal tumor of intermediate differentiation (PPTID), pineoblastoma (PB), papillary tumor of the pineal region (PTPR), and desmoplastic myxoid tumor of the pineal region, SMARCB1-mutant [26].

PC is a WHO grade 1 tumor characterized by slow growth and a favorable prognosis. PC can occur at all ages but is most frequently found in patients between 30 and 60 years [9, 10]. At the opposite end of the spectrum is PB, a highly aggressive CNS WHO grade 4 tumor of primitive neuroectodermal nature [24]. PBs predominantly affect young children, particularly those under 2 years of age. DNA methylation profiling has identified five distinct molecular subgroups (PB-GRP1A, PB-GRP1B, PB-GRP2, PIN-RB, and PB-MYC/FOXR2), which are characterized by distinct molecular profiles, genetic drivers, demographic features, and clinical outcomes [5, 10, 12, 35].

PPTID presents a diagnostic challenge due to its intermediate histological appearance. It is defined as a neoplasm with a diffuse or vaguely lobulated architecture [26]. Small in-frame insertions in the *KBTBD4* gene are characteristic for PPTID and are found in up to 80% of cases [21, 23, 39, 42]. PPTIDs are classified as WHO grade 2 to 3 tumors. PPTID comprises two distinct methylation groups, although correlation with histological or clinical parameters remains unclear [10, 25, 31].

PTPR is a rare neuroepithelial tumor [26]. It is classified as a WHO grade 2 or 3 neoplasm and can occur in a wide age range, from 15 months to 67 years [10]. Histologically, PTPRs are characterized by an epithelial-like, papillary growth-pattern, and the presence of rosette-like structures [10]. Immunohistochemically, they show a strong expression of S100, cytokeratin, SPDEF, vimentin, and neuron-specific enolase (NSE) [15]. In contrast to pineal parenchymal tumors, CRX expression is consistently absent [34]. Three distinct DNA methylation subgroups have been identified, which appear to vary in genomic CNV load as well as progression-free survival (PFS) [15, 40].

Finally, two very rare tumor types have been described in the pineal gland. One of them was a desmoplastic myxoid tumor of the pineal region, *SMARCB1*-mutant (DMT *SMARCB1*-mutant) [26, 28], and the other presented molecular features known from WNT medulloblastoma [24].

Together, the diagnosis of pineal region tumors remains challenging due to their overlapping histopathological features and molecular diversity. Nevertheless, genome-wide DNA methylation profiling has represented a major advance in this field, not only in the subclassification of tumor entities but also in improving diagnostic accuracy.

Here, we describe a previously unrecognized tumor type arising in the pineal region (*n*= 27) characterized by a unique and highly consistent genome-wide DNA methylation profile. This epigenetic signature does not correlate with any established CNS tumor class and is distinct from all currently recognized pineal tumor entities.

## Materials and methods

### Patient cohort

Our cohort consisted of 27 pineal tumors from 27 patients. Inclusion criteria were the availability of DNA methylation data and basic clinical and neuropathological annotations; cases with insufficient molecular data quality were excluded from further analyses. The cases included in this study were accessed through the classifier database following upload to www.molecularneuropathology.org. Corresponding clinical and histopathological data were obtained from the submitting institutions, with additional material or information retrieved through direct communication with the referring neuropathology centers.

The reference samples included 166 previously published cases of pineal tumors [3, 22, 24, 36, 37, 40], along with 76 previously unpublished reference cases. All reference cases matched a tumor group as defined by the CNS Tumor Methylation Classifier (v12.8) with a calibrated score of 0.9 or higher.

### Histology and immunohistochemistry (IHC)

We performed hematoxylin and eosin (H&E) stains on paraffin sections of all available tumors, following standard procedures. The evaluation was conducted per the 2021 WHO classification of tumors of the CNS [26].

The tumor cell proliferation rate was semi-quantitatively assessed using the Ki-67 labelling index. IHC was performed using a Ventana System (Roche) according to the manufacturer’s specifications. IHC analyses were conducted using antibodies against OTX2 (Thermo Fisher, 1H12C4B5, 1:2000), cytokeratin 18 (Dako, GA618, ready-to-use), synaptophysin (Dako, M7315, 1:500), CRX (Santa Cruz, sc377138, 1:200), desmin (Dako, M0760, 1:200), chromogranin A (Dako, M0869, 1:800), SPDEF (OriGene, TA324209, 1:200), and ATRX (Atlas antibodies, HPA001906, 1:300). Histological sections were also visually inspected for necrosis and calcifications.

### DNA methylation profiling

DNA was isolated from formalin-fixed, paraffin-embedded (FFPE) tissue using the ReliaPrepTM FFPE gDNA Miniprep System (Promega) according to the manufacturer’s instructions. About 100–500 ng of DNA were bisulfite converted (EZ DNA Methylation Kit; Zymo Research). Afterwards, the DNA Clean & Concentrator-5 (Zymo Research) and the Infinium HD FFPE DNA Restore Kit (Illumina) were employed to clean and restore the converted DNA. Finally, samples were analyzed using one of the following Illumina arrays on an iScan device (Illumina): HumanMethylation450 BeadChip array, Infinium MethylationEPIC BeadChip Kit, or Infinium MethylationEPIC v2.0 Kit (all Illumina). We obtained the affiliation predictions for established tumor types of the CNS by using the CNS Tumor Methylation Classifier [3], available at https://app.epignostix.com. This tool evaluates the global DNA methylation pattern using a random forest classifier. From this supervised classifier score, an entity-adjusted, so-called calibrated score is returned that intends to indicate the similarity between the sample and the underlying reference dataset for each trained type. We used the classifier version v12.8 for our analyses and considered the cut-off value of ≥0.9, as suggested by the authors of the algorithm, to indicate a significant match. Our reference samples from the Capper et al. [3] dataset encompass cases used for the v11b2 version of the German Cancer Research Center (DKFZ) CNS Tumor Methylation Classifier, which were then reclassified using the v12.5 version.

### Bioinformatics

Data processing was performed in R (v4.3.2 in Microsoft Windows). Our tumor samples and the reference cohort methylation data were processed using the “minfi” package in version 1.48.0 [19]. This package estimates and removes the background noise from out-of-bound probes, while the dye-bias normalization uses a subset of the control probes [38]. Detection *p* values were below the 0.01 cut-off, point and duplicate cases were recognized and removed using genotype matching. The beta values were calculated using the ‘getBeta’ function, followed by filtering probes using the ‘rmSNPandCH’ function of the “DMRcate” package [29]. This function eliminated any probes containing SNPs within two nucleotides of the CpG sites, probes associated with the sex chromosomes and sites not on the BeadChips were discarded. For visualization, the 10,000 most variable methylation probes were chosen based on standard deviation. The package “Rtsne” (v0.17) [20], dims = 2, perplexity = 15, and iterations = 10,000 was used to create *t*-SNE and Heatmap plots. Losses and amplifications in the copy number profiles were analyzed using the packages “conumee” (v1.36.0) and “conumee2.0” (v2.0) [6]. To identify relevant heterozygous losses or gains, a threshold of ±0.4 was chosen and a cut-off of ±0.8 was used to determine homozygous losses or amplifications. All of our samples’ copy-number variation (CNV) profiles were visually examined for validation.

### Next-generation sequencing

RNA and DNA were extracted from FFPE tumor tissue and analyzed using the TruSight Oncology 500 Assay (TSO500, Illumina) according to the manufacturer’s instructions. The sequence data were aligned to the human reference genome hg19 for DNA analyses and hg38 for RNA analyses. RNA sequencing data were additionally evaluated for gene fusions. The variants were annotated using information from the 1000 Genomes Project, dbSNP, ClinVar, and COSMIC. Only variants with an allele frequency ≥ 5% and a total target coverage ≥40× were considered for further analysis. Variants classified as benign, likely benign, or representing known polymorphisms were excluded from further evaluation.

### Single-nucleus RNA sequencing (snRNA-seq)

SnRNA-seq was performed on formalin-fixed paraffin-embedded (FFPE) tissue using the Next-GEM Chromium Single Cell Flex Gene Expression Assay (10× Genomics). Cells were isolated according to the protocol *“Isolation of Cells from FFPE Tissue Sections for Chromium Fixed RNA Profiling”* (Document Number CG000632 Rev B, 10× Genomics) using a gentleMACS Octo Dissociator (#130–134-029, Miltenyi Biotec) workflow. FFPE tissue scrolls were dissociated with an enzyme mix containing Liberase TH (#5401151001, MilliporeSigma). The dissociated nuclei suspension was stained with 4′,6-diamidino-2-phenylindole (DAPI, 1:5000). A BD FACSAria-Fusion™ Flow Cytometer was used to perform fluorescence-activated cell sorting of DAPI-positive nuclei to minimize erythrocytes and debris contamination. Probe hybridization was performed in-house in the University Medical Center Hamburg-Eppendorf (Germany) using the *Chromium Fixed RNA Human Transcriptome kit* PN-1000476 (4 rxns × 16 BC) according to the protocol “Chromium Fixed RNA Profiling Reagent Kits for Multiplexed Samples” (Document Number CG000527 Rev D, 10× Genomics). In total, four PINNED tumors and one PB sample were processed. Library preparation was performed at the UKE Single Cell Core Facility (Hamburg). The resulting multiplexed libraries were sequenced by CeGaT (Tübingen, Germany). We aimed for a target recovery of 80,000 cells per library. Cell Ranger v8.0.1 was used to generate feature-barcode matrices using the cellranger ‘multi’ command with default parameters. The GRCh38 reference genome (refdata-gex-GRCh38-2020-A) and Chromium Human Transcriptome Probe Set v1.0.1 were used for alignment and quantification. Cell Ranger sequencing metrics reports were reviewed to assess sample and sequencing quality.

Subsequent analyses were performed in R (v4.3.2). Unless otherwise specified, snRNA-sequencing data were analyzed using the ‘Seurat’ package (v4.4.0) together with ‘SeuratObject’ (v4.1.4) [14]. The cells were retained following quality control if they contained between 200 and 5000 detected features. Features detected in fewer than 10 cells per sample and cells with more than 5% mitochondrial transcript content were excluded. Pseudobulk expression profiles for each sample were generated using the ‘AggregateExpression’ function. UMAP projections of PINNED tumor cells into the reference developmental pineal gland have been performed as previously described [12].

## Results

**Fig. 1:**
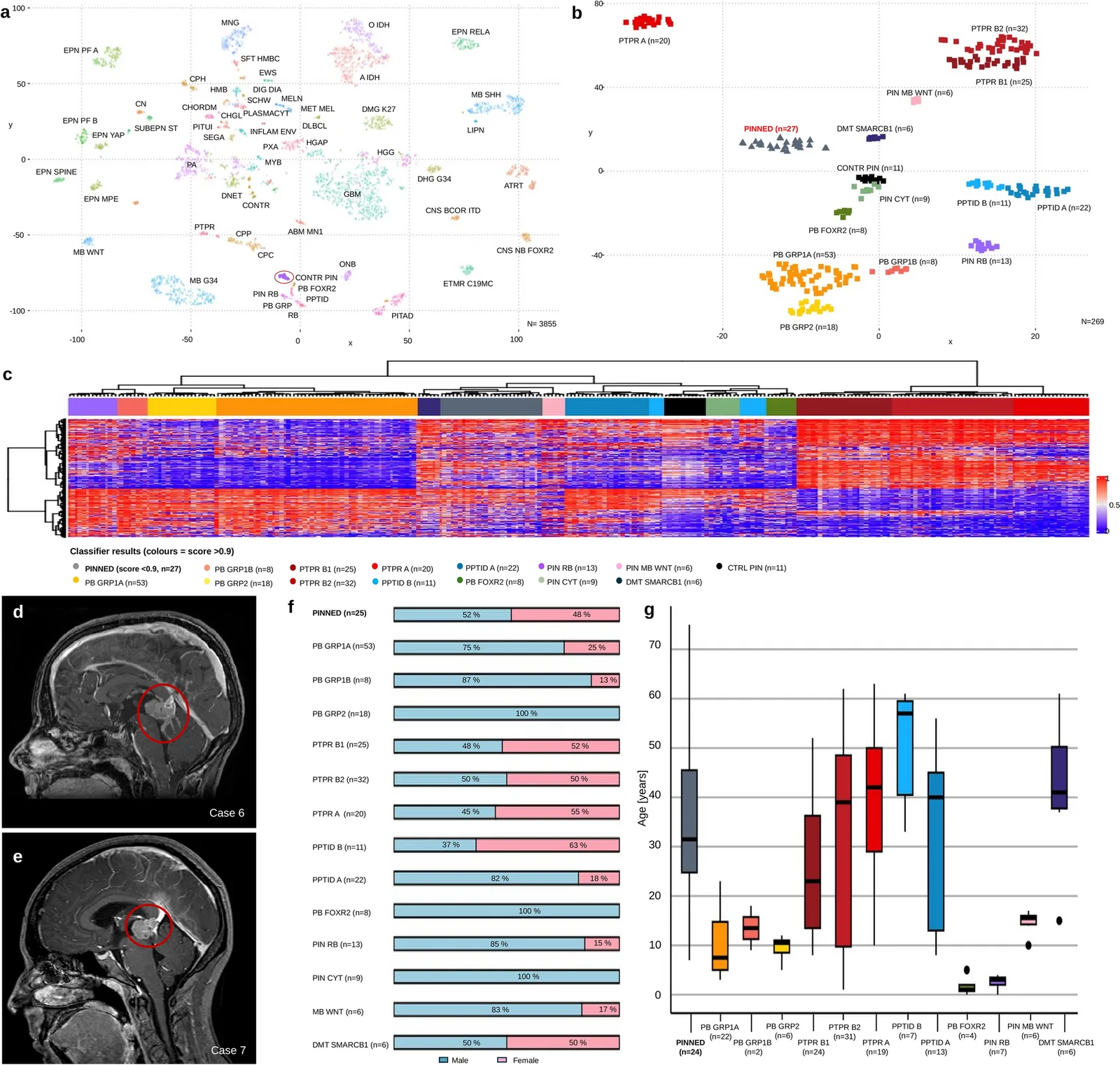
*t*-SNE plots illustrating our novel pineal subgroup (**a**, **b**) in comparison to reference cases. **a** shows the unclassified pineal tumors encircled in red as a distinct cluster from the defined Capper reference groups of 2018 [v12.5]. **b** and **c** display these ‘*Pi*neal *N*eoplasm with *N*euroendocrine and *E*pithelial *D*ifferentiation’ (PINNED) alongside a selection of 242 pineal reference cases obtained from published work [22, 24, 36, 37, 40] and from unpublished cases. **d** and **e** show the MR images of cases #6 and #7 with tumors encircled in red. **f** and **g** feature bar graphs depicting the sex (**f**) and age (**g**) distribution across the subgroups portrayed in **b** and **c**, with the median age denoted by a black line

We investigated a cohort of 27 tumors that clustered separately from all reference pineal tumor samples based on global DNA methylation profiling, as visualized by *t*-SNE and hierarchical clustering. Furthermore, these tumors did not match any previously described tumor entity using the CNS Tumor Methylation Classifier (score < 0.9, version v12.8, Fig. 1a–c, Suppl. Table 1). The tumors were provisionally named ‘*Pi*neal *N*eoplasm with *N*euroendocrine and *E*pithelial *D*ifferentiation’ (PINNED, see further below). To further characterize these tumors, we assessed their anatomical location, as well as the patients’ age distribution and sex balance. As shown in representative examples, tumors were located within the pineal region (Fig. 1d, e). The patients had a mean age of 35 years (range: 7–75 years), and 52% were male (Fig. 1f, g). Only for less than half of the patients, gross total resections were achieved. Available follow-up data showed death of disease in four cases and progressive disease in an additional seven cases with a 5-year progression-free survival below 25% (Suppl. Table 1). Together, these results describe a pineal gland–localized tumor with a distinct DNA methylation clustering pattern occurring in patients with a broad age distribution, a balanced sex distribution, and a clinical course that may include progressive disease or even death.

**Fig. 2:**
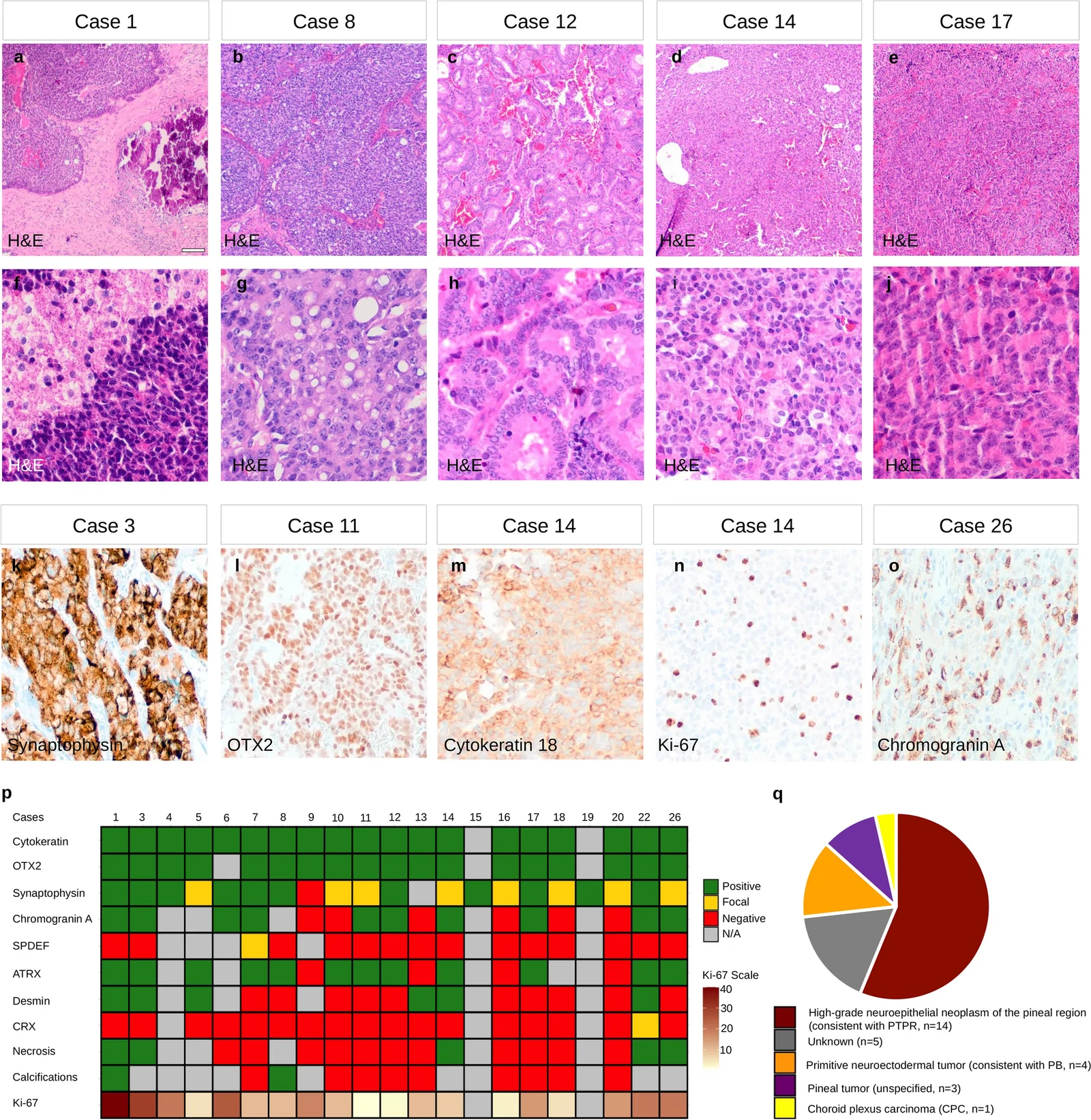
Histological diversity in PINNED tumors. **a–j** depict H&E stains from five representative cases (#1, #8, #12, #14, and #17). **k–o** show immunohistochemical stains from cases #3, #11, #14, and #26. **p** shows a heatmap summarizing all immunohistochemical findings; “N/A” indicates stains not available for the respective case. Positivity for Ki67 is given in percent of tumor cells. **q** shows the distribution of histological diagnoses signed out by referring institutions. The scale bar in **a** corresponds to 100 µm for panels **a–e** and to 20 µm for panels **f–o**

In the next step, we took a closer look at the histopathological and immunohistochemical profile of the tumors. Figure 2a–j shows five representative examples that reveal a relatively heterogeneous morphology throughout the cohort (Fig. 2a–e: low power; Fig. 2f–j: high power). Apart from overt mitotic figures, cases recurrently demonstrated solid architecture with sharp demarcation from the adjacent nervous tissue as well as calcifications (2/11 tumors (18%), Fig. 2a), necrosis (5/16 tumors (31%), Fig. 2f), or nested-growth pattern (Fig. 2b). Signet-ring morphology (Fig. 2g) that occurred in some of the cases was diffusely visible in the tumor tissue without overt relation to distinct architectures. Further features included packed tubes with true lumina resulting in a “pseudoglandular” architecture (Fig. 2c, h), epithelioid cytology (Fig. 2d, i), or densely-packed undifferentiated tumor tissue (Fig. 2e, j). Multinucleated, vacuolated cells or rhabdoid cells were visible in some specimens throughout the tumor with rhabdoid cells showing some association with pseudopapillary structures. If present, true rosettes were frequent and in all parts of the tumor.

Immunohistochemically, the tumors displayed a characteristic profile with consistent expression of synaptophysin (Fig. 2k), OTX2 (Fig. 2l), cytokeratin 18 (Fig. 2m), with all of these markers being expressed even in less differentiated tumor cells. Mean Ki-67 proliferation index was 10% (Fig. 2n). Synaptophysin showed strong and diffuse expression in the cytoplasm of a fraction of tumor cells including signet-ring cells, either in single cells or as foci of positive cells, sometimes with a periluminal appearance. Other tumors showed widespread expression. OTX2 and cytokeratin 18 were positive throughout all cases. In contrast, chromogranin A was expressed in the majority of cases (Fig. 2o) and often presented as a dot-like or granular pattern. In some areas of the tumors, chromogranin A expression was stronger and broader. Other tumors presented with only single cells that showed a strong cytoplasmatic staining without apical enhancement or with tumor cells that harbored vacuoles displaying eccentric chromogranin A expression (signet-ring cells). A subset of tumors also showed single desmin-positive tumor cells in the tissue, whereas CRX expression was uniformly absent. Only one tumor demonstrated focal SPDEF expression, which had been described as a marker for PTPRs [15]; all remaining tumors were negative. Immunohistochemical analysis revealed loss of ATRX protein expression in 4/16 tumors (25%). Taken together, the histopathological characteristics indicate an epithelial and neuroendocrine histology with consistent expression of cytokeratin 18, synaptophysin, and OTX2, and frequent positivity for chromogranin A. A summary of all histological findings is provided in Fig. 2p. Because of these features, we provisionally named this tumor group: ‘*Pi*neal *N*eoplasm with *N*euroendocrine and *E*pithelial *D*ifferentiation’ (PINNED).

The original histopathological diagnoses assigned by the referring institutions are shown in Fig. 2q. Fourteen of 27 tumors (52%) had originally been diagnosed as PTPR or as a high-grade neuroepithelial neoplasm of the pineal region or other descriptions consistent with PTPR. Four tumors were described as a primitive neuroectodermal tumor, consistent with PB. Three tumors were only classified as pineal tumors, and one tumor was described to most closely resemble a choroid plexus carcinoma. In at least four cases, the diagnosis of a metastatic neuroendocrine tumor in the pineal region has been discussed.

**Fig. 3:**
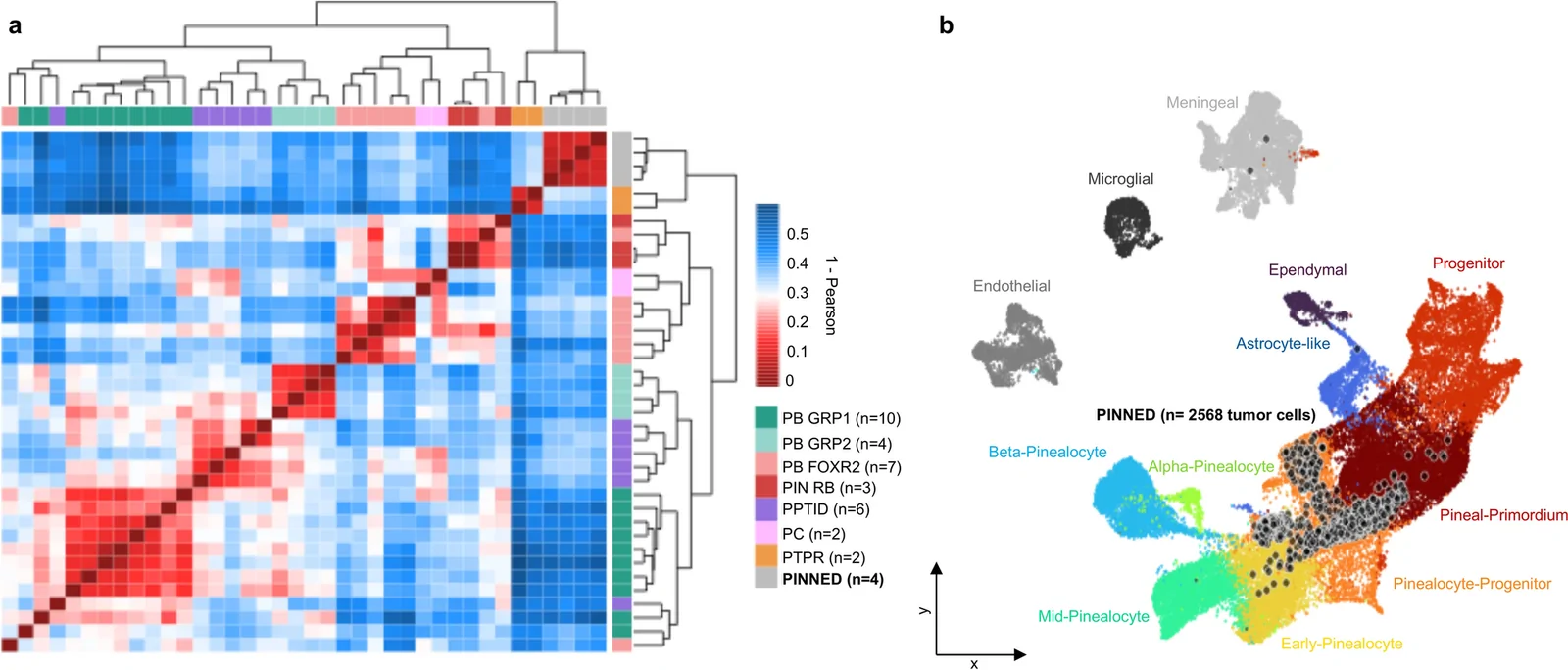
Pseudobulk analyses of snRNA-seq data from tumor cells of four PINNEDs tumor cells compared with other pineal tumor types in a similarity heatmap (**a**). UMAP analysis of the same four PINNED tumors using single-cell data from normal murine pineal development [12] demonstrate the highest similarities to pinealocyte-progenitors (**b**). PINNED tumor cells (*n* = 2568) are shown in larger black dots

To understand the transcriptome of PINNED, compare the transcriptome of these tumors with other tumors of the pineal region, and to better understand the cellular origin of the tumors, we generated and analyzed snRNA-seq data from four PINNED tumors. When comparing pseudobulk neoplastic nuclei data with 34 other pineal and pineal region tumor samples [12] based on gene co-expression patterns visualized in a similarity heatmap, PINNED demonstrated the greatest transcriptomic similarity to PTPR but formed a distinct tumor group (Fig. 3a). Projection of the transcriptomic data onto an atlas of the developing murine pineal gland [12] revealed the closest proximity of PINNED tumor cells with pinealocyte-progenitors (Fig. 3b).

**Fig. 4:**
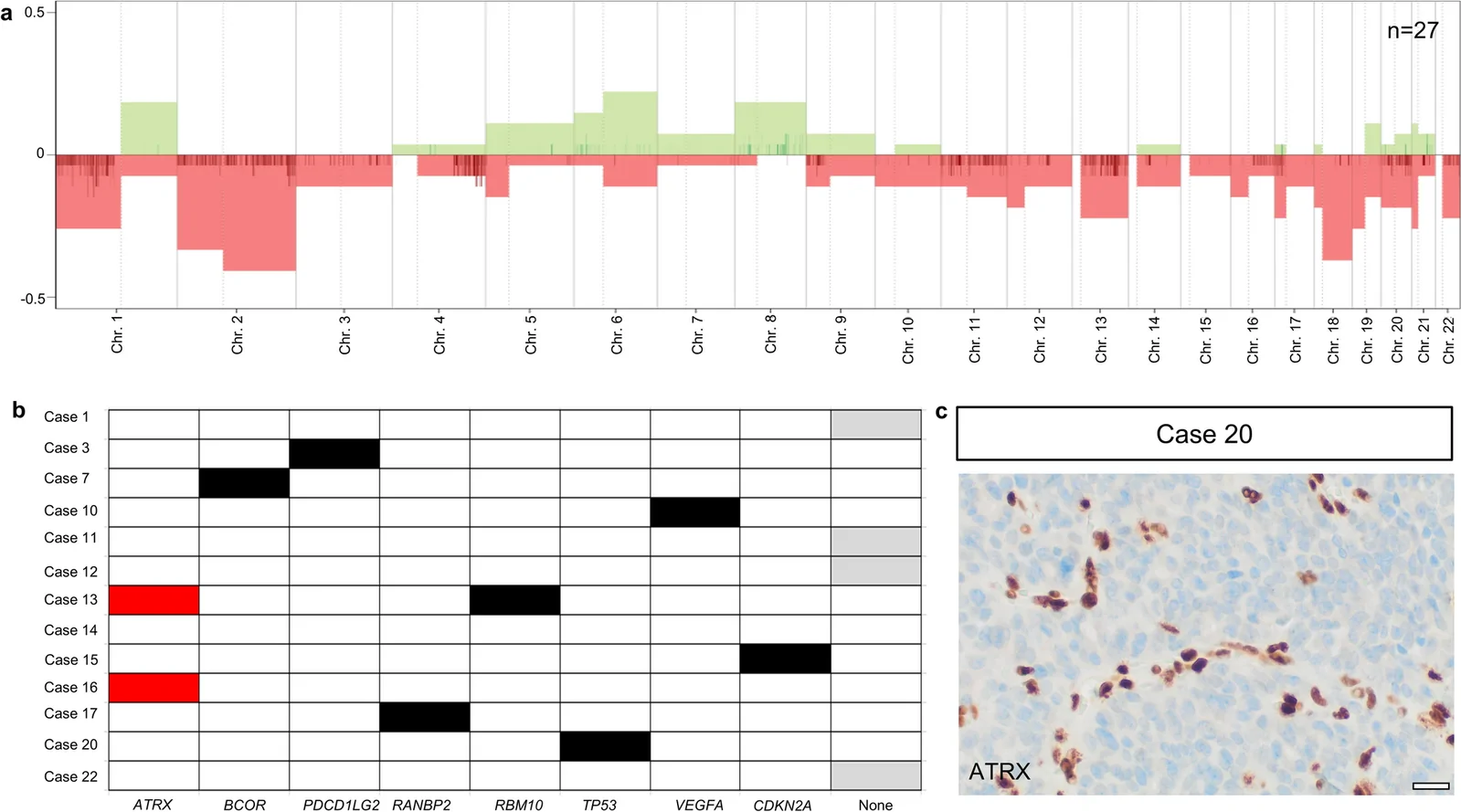
**a** Overview of chromosomal gains and losses seen in PINNED tumors. The plot shows a cumulative copy-number profile as inferred from global DNA methylation profiling data. **b** provides an overview of genetic alterations in PINNED. The plot shows all pathological mutations seen in the TSO500 panel sequencing of *n* = 13 cases. Mutations identified in multiple cases are highlighted in red, those detected in a single case are shown in black, and cases without any pathogenic variants are depicted in gray. **c** depicts IHC for case #20 using antibodies against ATRX. Non-neoplastic vessel and immune cells serve as internal positive control. The scale bar represents 20 µm

We finally sought to identify potential genetic drivers of these tumors. Genomic profiling revealed multiple copy number alterations but no recurrent focal amplifications or deletions (Fig. 4a). Targeted sequencing using the TSO500 panel was performed in 13 tumors (Fig. 4b). Among these cases, *ATRX* mutations were identified in two tumors, both of which also exhibited loss of ATRX protein expression by immunohistochemistry (Fig. 4c). Furthermore, no clinically relevant gene fusions were detected among the 17 tumors analyzed by bulk RNA sequencing (data not shown). In conclusion, ATRX alterations were the most common molecular abnormality identified in our cohort, being present in 25% of tumors.

## Discussion

Our findings support the existence of a previously unrecognized tumor entity within the spectrum of pineal region tumors. Using the CNS tumor classifier, the tumors were consistently classified as “no match”, with the highest calibrated scores often assigned to medullomyoblastoma (MB_MYO) or Non WNT/Non SHH medulloblastoma (MB_G34) [3]. Notably, none of the tumors matched any established methylation class in the CNS Tumor Methylation Classifier (v12.8), suggesting they are not adequately represented in existing reference cohorts. Unsupervised analyses demonstrated that they indeed formed a clearly separate group within the spectrum of pineal region tumors but should epigenetically not be included in the spectrum of medulloblastoma. These clustering results remained consistent across multiple *t*-SNE and Heatmap visualizations and were robust with reference cohorts of different sizes. Together, these findings indicate that the tumors described here represent a biologically distinct group.

From a clinical perspective, the cohort also differs from several established pineal tumor entities. Our cohort showed a balanced sex distribution and a broad age range, with a median age of 35 years. This demographic profile is—within the range of pineal region tumors—more similar to PTPR [40, 41] than to PB, which predominantly affects young children and often shows a male predominance [10, 40]. Although our cohort is limited in sized, it appears that 5-year progression-free survival is lower than both for PTPRs and PBs.

Additionally, many tumors in our cohort were originally classified by neuropathologists as PTPR, most likely due to their focal papillary growth pattern or the presence of occasional rosettes and epithelioid cytology. This similarity is underpinned by the absence of CRX expression, a specific marker of the retina and pineal gland [11], which is typically not expressed in PTPR [7], as well as strong expression of cytokeratin 18. However, several findings argue against a classification as a PTPR subgroup. First, our cohort consistently showed convincing synaptophysin and chromogranin A expression, and mostly lacked SPDEF expression, which was reported in 79% of PTPRs by Heim et al. [15]. Furthermore, upon central review, the tumors displayed distinctive histopathological features. Compared with PTPRs, the tumoral architecture was mostly solid. Papillary structures were absent or only focal, and less well-delineated. Importantly, despite partial morphological overlap, the combined immunohistochemical and histopathological findings support distinction from the currently recognized PTPR tumor entity.

Although these samples showed their greatest transcriptional similarity to the two available PTPR reference tumors, Pseudobulk snRNA-seq analysis of four PINNEDs demonstrated that they diverged before branching into any established PTPR subgroup when looking at an unsupervised hierarchical clustering.

Furthermore, the copy-number profiling revealed no recurrent chromosomal gains or losses, in contrast to PTPR, which exhibited chromosome 10 loss on all analyzed tumors, as well as recurrent losses of chromosomes 3 and 22q and gains of chromosome 8p [15]. Similarly, recurrent mutations observed in PTPR, PB, or PPTID, such as variants in *PTEN, RB1, DROSHA*, or *KBTBD4,* were absent in PINNED. Notably, the loss of ATRX expression was identified in four of 16 analyzed tumors, representing the most frequent molecular alteration in our cohort. ATRX alterations have previously been reported in two pineal tumors diagnosed as PPTID that were independent from our cohort [27]. However, such alterations have not emerged as a recurrent defining feature of established pineal tumor entities, making their relatively high frequency in our cohort particularly intriguing.

The presence of secretory and neuroendocrine architectural features, including nested growth patterns, rosette formation, and signet-ring cells, suggests neuroendocrine differentiation in these tumors. The primary intracranial neuroendocrine neoplasms are exceedingly rare, with only a limited number of cases reported in the literature [2, 5, 8, 13, 16, 18, 33]. Notably, both previously reported neuroendocrine tumors of the pineal gland demonstrated expression of synaptophysin, chromogranin A, and cytokeratin [5, 13], findings that overlap noticeably with the phenotype observed in our cohort. Distinguishing PINNEDs is crucial, as their histological appearance and cytokeratin expression may also resemble metastatic carcinoma, making this an important differential diagnosis for adult patients.

Despite these neuroendocrine and epithelial features, cell-of-origin analyses using a murine pineal gland reference dataset indicated a clear relationship to pineal gland development, with the strongest similarities observed to pinealocyte progenitor cells [12]. A similar observation has been made for PBs of different subtypes, whereas PTPRs show transcriptional similarities to the pineal primordium, further strengthening the notion that PINNED are most likely not a subset of PTPRs. The expression of CRX is neither observed in the pineal primordium nor in PTPRs. On the other hand, pinealocyte progenitor cells, although to a lesser extent than mature pinealocytes, demonstrate CRX expression, and it remains unclear why CRX is rather strong in PB but absent in PINNED although both entities show the highest similarities to pinealocyte progenitor cells. Nevertheless, our findings provide evidence that PINNED may arise from within the pineal developmental lineage and suggest that our cohort is a pineal tumor entity rather than an ectopic neoplasm located in the pineal region.

This study has several limitations. Although we report a relatively large cohort for a rare and previously unrecognized methylation class, the total number of cases remains limited and independent external validation cohorts are warranted. Moreover, the genetic drivers underlying tumor development remain largely unclear and the clinical course and long-term prognosis of affected patients cannot yet be defined due to limited follow-up data. While the integrated epigenetic, histopathological, and molecular data consistently support the identification of a distinct tumor entity, further studies in larger and independent cohorts will be necessary to confirm the reproducibility of these findings and to better define the full biological, genetic, and clinical spectrum of this cohort.

In conclusion, our findings support the existence of a novel type of pineal region tumor with distinct clinical, histopathological, and molecular features that we provisionally named PINNED.

## Supplementary Information

Below is the link to the electronic supplementary material.

Supplementary file1 (XLSX 16 KB)

## Data Availability

DNA methylation data are available via GEO accession number GSE338335.
